# Complications associated with open reduction and internal fixation for adult distal humerus fractures: a multicenter retrospective study

**DOI:** 10.1186/s13018-022-03292-1

**Published:** 2022-08-31

**Authors:** Soo-Hong Han, Jin Sung Park, Jong Hun Baek, Segi Kim, Ki Hyeok Ku

**Affiliations:** 1 Department of Orthopedic Surgery, CHA Bundang Medical Center, CHA University School of Medicine, 59, Yatap-ro, Bundang-gu, Seongnam-si, Gyeonggi-do, 13496 Republic of Korea; 2 Division of Hand & Wrist Surgery and Microsurgery, Department of Orthopedic Surgery, Yeson Hospital, 206, Bucheon-ro, Bucheon-si, Gyeonggi-do, 14555 Republic of Korea; 3 Department of Orthopedic Surgery, Kyung Hee University Hospital, Kyung Hee University School of medicine, 23 Kyunghee-daero, Dongdaemun-gu, Seoul, 02447 Republic of Korea; 4 Department of Orthopedic Surgery, Kyung Hee University Hospital at Gangdong, Kyung Hee University School of Medicine, 892, Dongnam-ro, Gangdong-gu, Seoul, 05278 Republic of Korea

**Keywords:** Distal humerus fracture, Plate fixation, Orthogonal plate, Parallel plate, Single plate, Complication

## Abstract

**Background:**

Open reduction and plate fixation are the preferred treatment options for most distal humerus fractures in adults. However, it is often challenging for orthopedic surgeons because of the complex anatomy and the difficulty in achieving stable fixation. This multicenter study aimed to analyze the complication types and rates of patients with distal humerus fractures treated with open reduction and plate fixation, and compare the results with those found in the literature. In addition, we describe the clinical outcomes.

**Methods:**

This retrospective multicenter study was conducted between September 2001 and March 2021 and included data from four hospitals. In total, 349 elbows underwent surgical treatment at these hospitals during the study period. Patients > 17 years of age who were treated by plate fixation were included, and patients who were treated by other fixation methods were excluded. A total of 170 patients were included in the study. The following types of complications were investigated: (1) nerve related; (2) fixation and instrument related; (3) osteosynthesis related; (4) infection; and (5) others.

**Results:**

The following complications were found: (1) 26 (15.3%) cases of postoperative ulnar nerve symptoms; 4 (2.4%) of postoperative radial nerve symptoms; (2) one (0.6%) case of screw joint penetration and screw loosening; and eight (4.7%) cases of hardware removal due to instrument skin irritation; (3) seven (4.1%) cases of nonunion; (4) two (1.2%) and four (2.2%) cases of superficial and deep infection, respectively, and seven (3.9%) cases of wound complication; and (5) 37 (21.8%) cases of heterotrophic ossification, 79 (46.5%) cases of elbow stiffness (did not achieve functional range of motion [ROM]), and 41 (24.1%) cases of osteoarthritis over Broberg and Morrey Grade I.

Paradoxically, the postoperative ulnar nerve symptoms were more frequent in the prophylactic ulnar nerve anterior transposition group. However, this difference was not statistically significant (*p* = 0.086). The mean ROM was 123.5° flexion to 9.5° extension. The average Disabilities of the Arm, Shoulder and Hand (DASH) score was 14.5 ± 15.6.

**Conclusions:**

Open reduction and plate fixation for distal humeral fractures is a reasonable treatment option with acceptable complication rates and favorable clinical outcomes. Surgeons must be vigilant about ulnar nerve complications.

*Level of Evidence *Therapeutic Level III.

**Supplementary Information:**

The online version contains supplementary material available at 10.1186/s13018-022-03292-1.

## Background

Distal humerus fracture in adults is one of the most challenging injuries to treat for orthopedic surgeons. Complex regional anatomy containing neurovascular, articular comminution, and a limited point for secure fixation makes it challenging for surgeons to achieve anatomical reduction and stable fixation [1]. Some orthopedic surgeons are not familiar with distal humeral fractures because of their relatively low incidence in adults [2].

Most distal humeral fractures in adults must be treated surgically. Although total elbow arthroplasty is a viable treatment option, open reduction and plate fixation are the preferred treatments for adult distal humerus fractures.

The outcome of distal humerus fracture has improved with advancements in implants and surgical approaches, with good to excellent results in approximately 85% of older patients [3, 4]. However, some studies have reported high complication rates recently [5]. Distal humerus fracture itself and surgical open reduction and internal fixation (ORIF) can cause nerve injury, screw joint penetration, nonunion, infections, heterotrophic ossification, and elbow stiffness [6].

Efforts have been made to identify the types and rates of complications after plate fixation for distal humeral fractures. However, the study population was < 50 patients in most of the studies [4, 5, 7–9]. Therefore, this multicenter study was conducted to investigate the complications of distal humerus plate fixation. This study aimed to analyze the complication types and rates of distal humerus fractures treated with open reduction and plate fixation, and compare the results with those found in the literature. In addition, we describe the clinical outcomes.

## Methods

### Study design and patient selection

This retrospective multicenter study reviewed patient records from September 2001 to March 2021, approved by the local institutional review board (KHUH 2021-04-037-004). Data from patients treated by seven hand or shoulder surgeons performing elbow surgery at four university hospitals were retrieved for analysis in this study. Two independent orthopedic surgeons from each of the four hospitals (eight orthopedic surgeons in total) were responsible for collecting the data. Conflicting data were sent back to each hospital and reviewed. Data collection was conducted over two months.

**Fig. 1:**
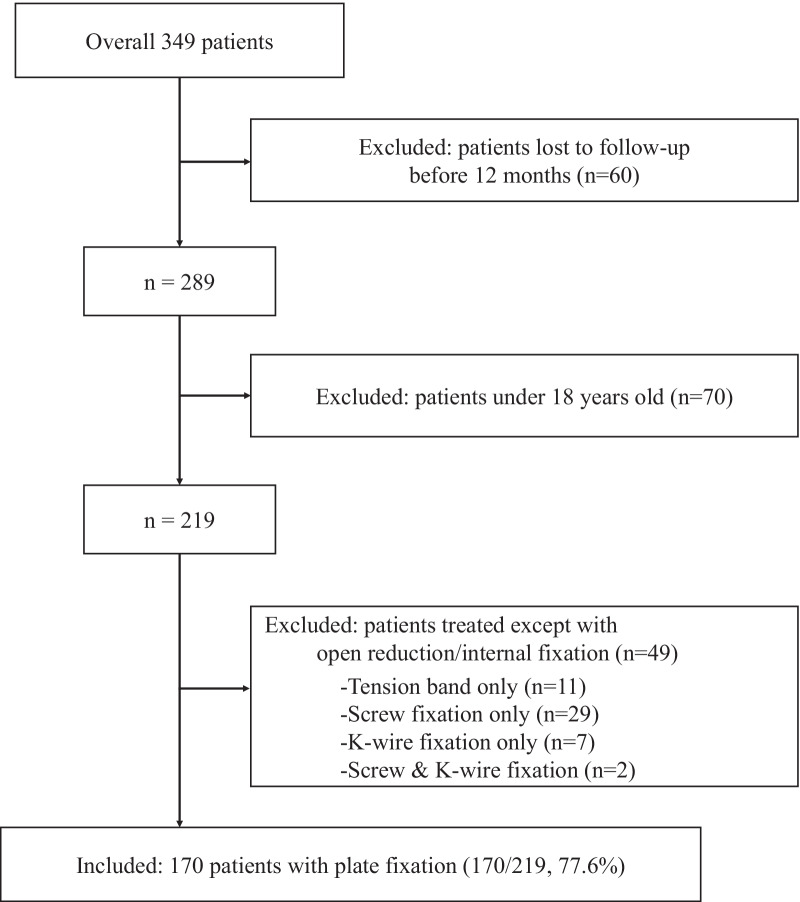
Study flow diagram of participants

During the study period, 349 distal humeral fractures were diagnosed and treated operatively. We excluded patients with insufficient information due to incompleteness or loss of medical records, and those who were not followed-up until the surgeon determined the fracture union or did not follow-up 12 months after the operation (60, 17.2%). Patients aged > 17 years were screened (70, 24.2% excluded). Thus, 219 patients were eligible for further screening. Only patients with distal humerus fractures treated with open reduction and plate fixation were included; those who underwent tension band fixation alone (11, 5.0%), K-wire fixation alone (7, 3.2%), screw fixation alone (29, 13.2%), and screw and K-wire fixation (2, 1%) were excluded. A total of 170 elbows from 170 patients were included in this study (77.6%; Fig. 1).

### Patient demographics and fracture characteristics

Medical charts and radiographs of each patient were reviewed at each hospital unit to determine patient demographics and fracture characteristics. Pre-operative radiography and computed tomography were used to determine fracture type according to the AO Foundation and Orthopaedic Trauma Association (AO/OTA) classification (types A-C). Fracture characteristics, such as degree of openness according to the Gustilo-Anderson classification, injury mechanism, injury side, time from injury to operation, and nerve or vascular injuries, were also documented. Intraoperatively, details of the surgical procedure were analyzed, particularly the surgical approach used, type of instrumentation applied, and associated procedures.

### Evaluation of complications and clinical outcome

Complications were categorized based on follow-up radiographs and medical chart reviews. Complications were categorized as follows: (1) Nerve related: postoperative ulnar nerve and other nerve symptoms, preoperative nerve symptoms; (2) Fixation and instrument related: screw joint penetration, reduction loss and fixation loosening, metal breakage, and skin instrumentation irritation; (3) Osteosynthesis related: nonunion, and mean union period; (4) Infection: superficial infection, deep infection, and wound complications; and (5) Others: heterotrophic ossification, elbow stiffness, osteoarthritis, and complex regional pain syndrome(CRPS) [10].

Postoperative nerve symptom diagnosis was based on physical examination or electrodynamic studies. Preoperative nerve symptoms were defined based on physical examination and patient symptoms. The number of patients with postoperative nerve symptoms requiring a secondary operation and the detailed surgical techniques used in secondary operations were also investigated.

Fracture union was determined when no distinct fracture gap was seen in any PA/lateral/oblique view of radiographs during follow-up. Nonunion was determined when there was no narrowing of the fracture gap on consecutive follow-up radiographs until six months after surgery [11–13].

Superficial infection was defined as the presence of local infection signs and treatment with antibiotics alone without surgical treatment. Deep infection was defined as the need for antibiotics and additional surgical debridement. Wound complications were defined as dehiscence of the wound or occurrence of skin defects.

If the elbow did not achieve a functional range of motion (130° flexion and 30° extension were satisfied), elbow stiffness was considered [14]. CRPS was diagnosed using the Budapest criteria [15, 16]. The Budapest criteria include persistent pain, sensory changes, vasomotor symptoms, sudomotor/edema, and motor/trophic changes.

As clinical outcomes, elbow flexion and extension range of motion(ROM) and DASH score were routinely checked in most patients at the last follow-up. Elbow extension and flexion were measured using a long handheld goniometer.

Finally, the complication rates and clinical outcomes in this study were compared with those reported in the literature. Among the available studies, we selected three. The first study evaluated the outcome of 32 complex distal humerus fractures treated with parallel plating, the second was a multicenter retrospective study with 289 cases, and third study was a recent study reported high complication rates [5, 7, 9]. Subgroup analysis of complication rates was also conducted using the subdivided fixation method and surgical approach.

### Statistical analysis

Descriptive statistical analyses were performed using the IBM SPSS software package (version 25.0, IBM, Armonk, NY, USA). Chi-square test and adjusted (age, sex) logistic regression analysis were used for dichotomous variables of prophylactic ulnar nerve anterior transposition and ulnar nerve symptoms. An adjusted (age, sex) generalized linear model was used for numerical data of elbow extension-flexion ROM to analyze the relationship of heterotrophic ossification with ROM.

## Results

The mean age of patients was 52.4 years (range, 18–83 years). Seventeen patients (10%) had open fractures, and 76 patients (44.7%) had experienced high-energy trauma, such as traffic accidents and falls (Table 1).

**Table 1:** Basic characteristics of study

Characteristics	170 patients (100%)
*Patient demographics*	
Mean age (years)	52.4 (SD 19.0)
Sex	
Male	49 (28.8%)
Female	121 (71.2%)
*Fracture characteristics*	
Injury mechanism	
High energy trauma	76 (44.7%)
Low energy trauma	94 (55.3%)
Fractured side	
Right	64 (37.6%)
Left	106 (62.4%)
Open Fracture	17 (10.0%)
AO/OTA classification	
13A2	39 (22.9%)
13A3	11 (6.5%)
13B1	5 (3.0%)
13B2	6 (3.5%)
13C1	24 (14.1%)
13C2	61 (35.9%)
13C3	24 (14.1%)
*Operation related*	
Pre-operative duration (days, SD)	5.6 (SD 8.7)
Mean operation time (minutes, SD)	164.4 (SD105.9)
Approach	
Olecranon osteotomy	88 (51.8%)
Triceps splitting approach	34 (20.0%)
Paratricipital approach	33 (19.4%)
Others/Unknown	15 (8.8%)
Type of plate	
Orthogonal plate	108 (63.5%)
Parallel plate	28 (16.5%)
Single plate	34 (20.0%)
Prophylactic ulnar nerve transposition	112 (65.9%)

*SD* Standard deviation

The posterior surgical approach was the most frequently used approach (91.2%) among the lateral, medial, and posterior approaches. In the posterior approach, the olecranon osteotomy approach was utilized the most. C2-type fractures were the most common (61 patients, 35.9%) in the AO/OTA classification (Table 1).

Elbow stiffness (46.5%), osteoarthritis (24.1%) and heterotrophic ossification (21.8%) were three most common complications. Except that, complication rates including all trivial minor complications were 30.6% (52 cases), and ulnar nerve complications accounted for half (26 cases, 15.3%) (Table 2).

**Table 2:** Complication comparison with previous reports

Complications (*n*, %) and functional outcome	Study group	Sanchez–Sotelo (*n* = 32) (2007)	Clavert (*n* = 289) (2013)	Patel (*n* = 43) (2020)	Authors (*n* = 170)
		Parallel plate Complex fracture	Above 65 Plate fixation	Contoured locking plate	Above 17 Plate fixation
Nerve related	Pre-operative nerve symptoms	1 (3%)	14 (4.8%)	8(18.6%)	11 (6.5%)
	Ulnar nerve symptoms: spontaneously recovered	2 (6%)	21 (7.3%)	3(7.0%)	15 (8.8%)
	Ulnar nerve symptoms: required operation			4(9.3%)	11 (6.5%)
	Other nerve symptoms		7 (2.4%)	1(2.3%)	4 (2.4%)
Fixation and instrument related	Screw joint penetration				1 (0.6%)
	Reduction loss and implant loosening		20 (7.0%)*		1 (0.6%)
	Metal breakage				0
	Skin implant irritation		38 (13.2%)		8 (4.7%)
Osteosynthesis related	Non-union of fracture	1 (3%)	18 (6.3%)	4(9.3%)	7 (4.1%)
	Mean union period (Week)				18.6 ± 11.2
	Non-union of Olecranon osteotomy	1/5 (20%)		1/22(5%)	1/82 (1.2%)
Infection	Superficial infection		14 (4.8%)		2 (1.2%)
	Deep infection	1 (3%)		4(9.3%)	4 (2.4%)
	Wound complications	2 (6%)	11 (3.8%)		7 (4.1%)
Others	Heterotrophic ossification	12 (38%)	73 (25.2%)	4(9.3%)	37 (21.8%)
	Elbow stiffness (cannot achieve 30°–130° of arc)	19 (59%)		8(18.6%)	79 (46.5%)
	Osteoarthritis (Broberg and Morrey Grade II or III)	2 (6%)	50 (17.3%)	4(9.3%)	41 (24.1%)
	Complex Regional Pain Syndrome				2 (1.2%)
Range of motion	Extension–flexion arc (°)	26–125°	24–121°		9.5–124°
DASH score		85(MEPS)	83(MEPS)		14.4 ± 15.2

*MEPS* Mayo elbow performance score

* Clavert at el didn’t separately discribe implant loosening and metal breakage. They reported 7% of Fixation failure rate retrospectively

### Nerve-related complications

Postoperative ulnar nerve symptoms were present in 26 patients (15.3%), and 15 patients (8.8%) recovered without reoperation. Most spontaneous recovery cases resolved within two years. Of the 11 patients with postoperative ulnar nerve symptoms who required ulnar nerve reoperation, one patient underwent emergent ulnar nerve decompression immediately after the initial surgery due to hematoma, four patients were treated with ulnar nerve decompression, while the other six were treated with ulnar nerve anterior transposition and decompression.

Postoperative radial nerve symptoms occurred in four patients (2.2%), and two patients recovered without surgery. In the other two patients who required radial nerve decompression, one patient was treated with radial nerve decompression with instrumentation removal. Another patient was treated with radial nerve decompression only.

Of the 11 patients with pre-operative nerve symptoms, three had ulnar nerve, six had radial nerve, one had median nerve, and one had triple nerve symptoms. In patients with preoperative nerve symptoms, only two radial nerve symptoms persisted postoperatively.

In 170 patients, simultaneous ulnar nerve anterior transposition was performed in 112 patients (65.9%) (Table 3). The proportion of postoperative ulnar nerve symptoms was higher in the ulnar nerve anterior transposition group. However, this difference was not statistically significant (*p* = 0.086).

**Table 3:** Nerve-related complications depending on whether simultaneous ulnar nerve prophylactic anterior transposition was present

Complication (*n*, %)	Prophylactic ulnar nerve transposition ( +) (*n* = 112, 100%)	Prophylactic ulnar nerve transposition (−) (*n* = 58, 100%)
*Nerve related*		
Pre-operative ulnar nerve symptoms	2 (1.8%)	2 (3.4%)
Ulnar nerve symptoms: spontaneously recovered	12 (10.7%)	3 (5.2%)
Ulnar nerve symptoms: required secondary operation	9 (8.0%)	2 (3.4%)
Median or radial nerve symptoms	2 (1.8%)	2 (3.4%)

### Fixation and instrument related, osteosynthesis-related complications

Screw joint penetration was identified in one patient (0.6%), and the screw was removed with reoperation. Reduction loss and implant loosening after fixation occurred in one patient, and refixation was performed one month after the initial fixation. There were no cases of metal breakage.

**Fig. 2:**
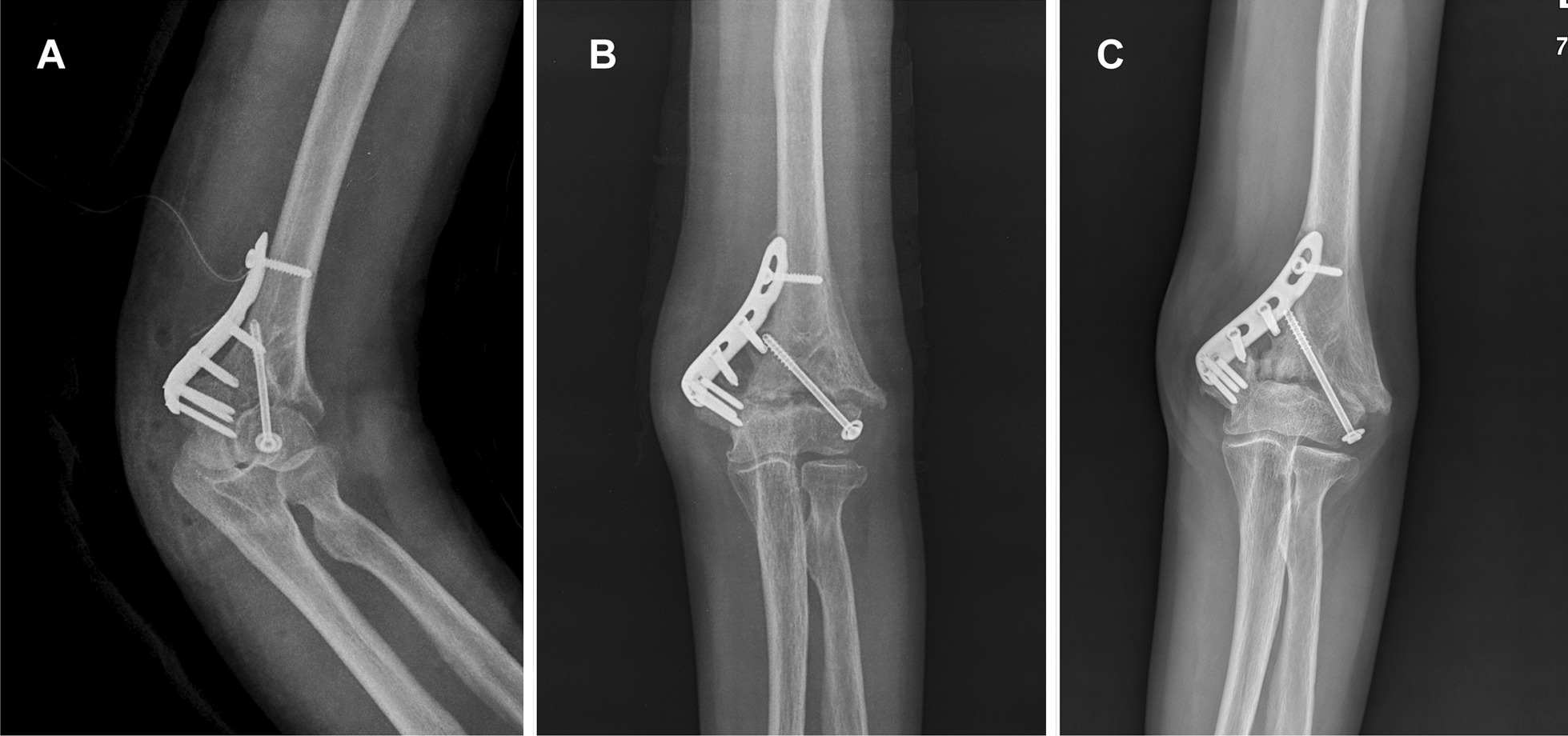
A 66-year-old female patient with painless nonunion. **A** Post-operative X-ray **B** At three months postoperatively, bone absorption was observed around the plate. **C** At two years after the operation, she could perform daily activities without pain, despite nonunion

**Fig. 3:**
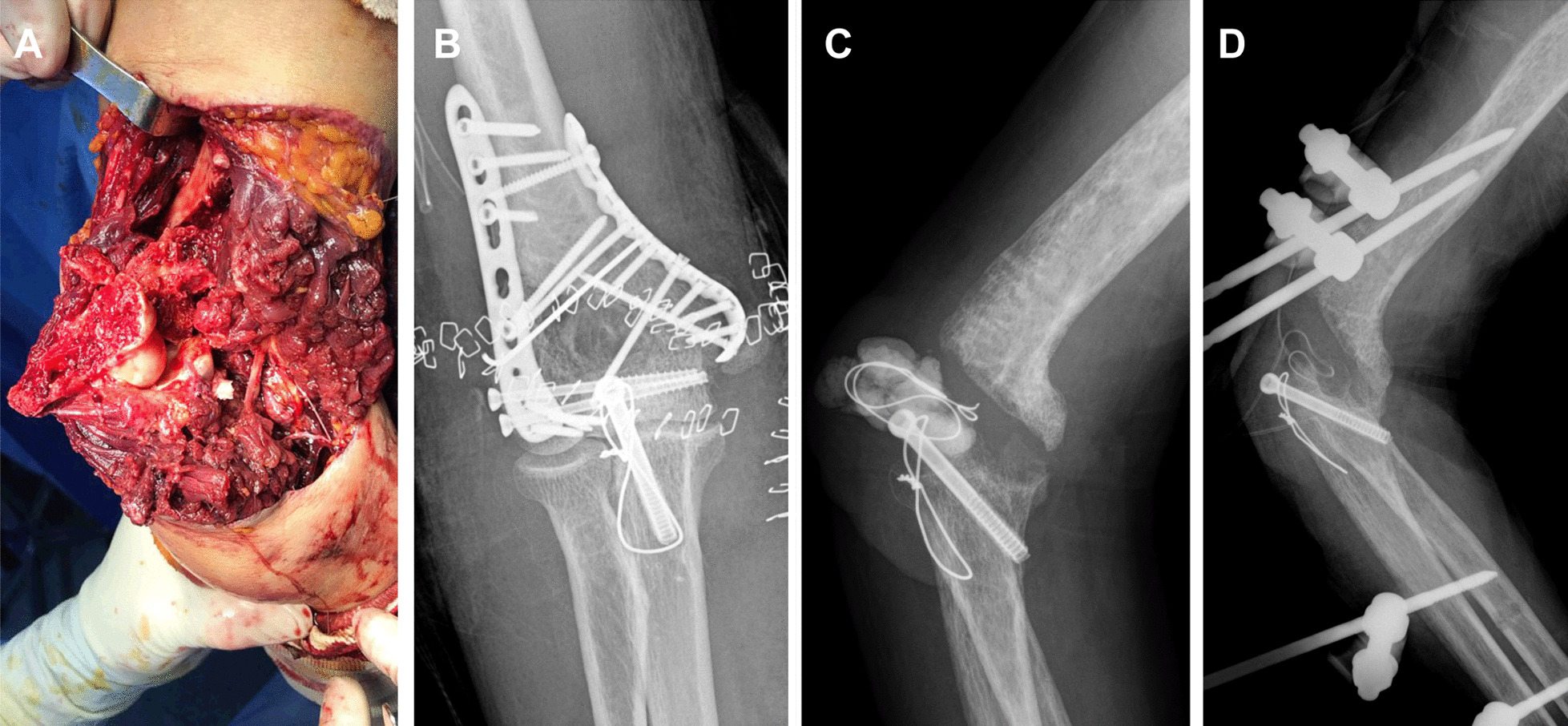
A 76-year-old male patient with a GA IIIA open fracture. **A**, **B** Open reduction and internal fixation were initially performed. **C** Due to uncontrolled infection, all implants were removed and anti-mixed cement was inserted. Joint was severely destructed. **D** After several debridement and external fixator application, the infection had not been controlled

Seven cases of nonunion were diagnosed. Two nonunion patients did not complain of discomfort even though they maintained daily living without a splint and refused osteosynthesis surgery (Fig. 2). Four patients underwent revision osteosynthesis with an autologous bone graft and achieved bone union. One patient with nonunion and infection did not achieve bone union until the time of data collection (Fig. 3). Nonunion rates were highest in single plate fixation (4 cases; 11.8%) and lowest in orthogonal plating (1 case; 0.9%).

Except for nonunion cases, the mean period for bone union was 18.6 ± 11.2 weeks.

### Infection and other complications

Among the four cases (2.4%) with deep infection, three had wound complications. Two wound complications were resolved with a local flap, and one patient with severe open fracture covered the soft tissue with a free flap (Fig. 3).

Thirty-seven patients (21.8%) had heterotrophic ossification. The extension-flexion ROM of 133 patients without heterotrophic ossification was 9.1 ± 11.4° to 125.4 ± 20.2°, and the ROM of 37 patients with heterotrophic ossification was 11.0 ± 12.6° to 116.6 ± 18.5°. The difference in flexion ROM was statistically significant (*p* = 0.003). The difference in extension ROM was not statistically significant (*p* = 0.197). Reoperation for heterotrophic ossification excision was not performed.

Two patients (1.2%) were diagnosed with CRPS. One patient improved with analgesics medication, and another patient improved with physical therapy.

### Clinical outcome and complication required reoperation

Of the 22 patients with major complications requiring urgent reoperation, 11 had ulnar nerve symptoms, one had radial nerve symptoms, three underwent deep infection debridement, one had deep infection and radial nerve symptoms, four had nonunion, one had reduction loss, and one had screw joint penetration.

The mean ROM was 123.5 ± 20.2° flexion to 9.5 ± 11.7° extension. The average DASH score was 14.4 ± 15.2.

### Comparison with previous studies and subgroup analysis

A comparison of our data with those of previous studies is presented in Table 2 [5, 7, 9]. Subgroup analysis according to fixation method and surgical approach is presented in Tables 4 and 5.

**Table 4:** Complication according to fixation method

Complication (*n*, %)		Orthogonal plate (*n* = 108, 100%)	Parallel plate (*n* = 28, 100%)	Single plate (*n* = 34, 100%)	Authors (*n* = 170)	
Nerve related	Pre-operative nerve symptom	8 (7.4%)	2 (7.1%)	1 (2.9.%)	11	
	Ulnar nerve symptom: spontaneously recovered	10 (9.3%)	4 (14.3%)	1 (2.9%)	15	
	Ulnar nerve symptom: required operation	10 (9.3%)	0	1 (2.9%)	11	
	Other nerve symptom	3 (2.8%)	1 (3.6%)	0	4	
Fixation and instrument related	Screw joint penetration	0	1 (3.6%)	0	1	
	Reduction loss and implant loosening	1 (0.9%)	0	0	1	
	Skin implant irritation	3 (2.8%)	2 (7.1%)	3 (8.8%)	8	
Osteosynthesis related	Non-union of fracture	1 (0.9%)	2 (7.1%)	4 (11.8%)	7	
	Mean union period (weeks)	19.1 ± 11.4	14.2 ± 4.6	20.6 ± 14.0	18.6 ± 11.2	
	Non-union of Olecranon osteotomy	1	0	0	1	
Infection	Superficial infection	2 (1.9%)	0	0	2	
	Deep infection	1 (0.9%)	1 (3.6%)	2 (5.9%)	4	
	Wound complications	4 (3.7%)	1 (3.6%)	2 (5.9%)	7	
Others	Heterotrophic ossification	26 (24.1%)	3 (10.7%)	8 (23.5%)	37	
	Elbow stiffness (cannot achieve 30° ~ 130° of arc)	48 (44.4%)	15 (53.6%)	16 (47.1%)	79	
	Osteoarthritis (Broberg and Morrey Gr II or III)	28 (25.9%)	4 (14.3%)	9 (26.5%)	41	
	Complex Regional Pain Syndrome	1 (0.9%)	0	1 (2.9%)	2	
Functional outcome						
Range of motion	Extension–flexion arc (°)	8° ~ 125°	15° ~ 112°	10° ~ 127°	9.5° ~ 124°	
DASH score		16.5 ± 15.6	15.1 ± 16.2	6.5 ± 10.1	14.4 ± 15.2

**Table 5:** Complication according to surgical approach

Complication (*n*, %)		Olecranon osteotomy (*n* = 88, 100%)	Triceps splitting (*n* = 34, 100%)	Paratricipital (*n* = 33, 100%)
Nerve related	Pre-operative nerve symptom	7 (8.0%)	2 (5.9%)	1 (3.0%)
	Ulnar nerve symptom: spontaneously recovered	10 (11.4%)	1 (2.9%)	4 (12.1%)
	Ulnar nerve symptom: required operation	6 (6.8%)	1 (2.9%)	3 (9.1%)
	Other nerve symptom	2 (2.3%)	1 (2.9%)	0
Fixation and instrument related	Screw joint penetration	1 (1.1%)	0	0
	Reduction loss and implant loosening	1 (1.1%)	0	0
	Skin implant irritation	5 (5.7%)	0	2 (6.1%)
Osteosynthesis related	Non-union of fracture	4 (4.5%)	1 (2.9%)	1 (3.0%)
	Mean union period (weeks)	19.0 ± 12.3	17.5 ± 11	18.7 ± 8.1
Infection	Superficial infection	0	2 (5.9%)	0
	Deep infection	1 (1.1%)	0	1 (3.0%)
	Wound complications	3 (3.4%)	2 (5.9%)	0
Others	Heterotrophic ossification	20 (22.7%)	9 (26.5%)	5 (15.2%)
	Elbow stiffness (cannot achieve 30° ~ 130° of arc)	44 (50%)	16 (47.1%)	11 (33.3%)
	Osteoarthritis (Broberg and Morrey Gr II or III)	26 (29.5%)	8 (23.5%)	4 (12.1%)
	Complex Regional Pain Syndrome	1 (1.1%)	1 (2.9%)	0
Functional outcome				
Range of motion	Extension–flexion arc (°)	11° ~ 122°	9.7° ~ 123.1°	4.1° ~ 128.4°
DASH score		16.0 ± 16.7	16.5 ± 15.0	14.3 ± 14.2

## Discussion

The results of this study were compared with those of three other retrospective studies [5, 7, 9]. Sotelo et al. reviewed 34 patients treated with parallel plating. They reported a 28% major complication rate that required reoperation, including 16% heterotrophic excision. Moreover, 15% of complications were reported without reoperation, including 6% of post-traumatic arthritis cases. Clavert et al. reported a multicenter retrospective study of 289 patients who were treated with plate fixation and were above 65 years of age. With the exception of 51.1% of traumatic osteoarthritis and 25.2% of heterotrophic ossification, implant cutaneous irritation (13.2%), and postoperative ulnar nerve symptoms (7.3%) were the most common complications. They did not include elbow stiffness as a complication rate. Patel et al. retrospectively reviewed the records of 43 patients. The overall complication rate was 60.5%, and the rate of complications requiring reoperation was 48.8%. They included 9.3% of heterotrophic ossification, 9.3% of post-traumatic arthritis, 9.3% of implant irritation, and 18.6% of joint stiffness as complications.

### Nerve-related complications

Postoperative ulnar nerve symptoms occurred in 15.3% of the patients in this study. Sotelo et al. and Clavert et al. reported postoperative ulnar nerve symptoms of 6% and 7.3%, respectively. Patel et al. reported that 9.3% of postoperative ulnar nerve symptoms were resolved through surgery and 7.0% of postoperative ulnar nerve symptoms resolved without surgery.

Paradoxically, postoperative ulnar symptoms occurred more frequently in the prophylactic anterior transposition group than in the group without anterior transposition (Table 3). Vazquez et al. reported that prophylactic ulnar nerve anterior transposition does not decrease the development of postoperative ulnar nerve symptoms [17]. Chen et al. compared two groups based on whether prophylactic ulnar nerve anterior transposition was performed. The incidence of ulnar neuritis was 33% in the anterior transposition ( +) group and 9% in the anterior transposition (−) group [18]. Chen et al. provided three explanations for this: (1) Additional handling and devascularization of the nerve during transposition, (2) Iatrogenic compression resulting from an overlying tight transposition, and (3) Inadequate proximal release of the arcade of Struthers or medial intermuscular septum.

To avoid ulnar nerve complications, combined anteromedial and anterolateral approach and plating could be a good treatment option without ulnar nerve neurolysis or anterior transposition[19, 20]. Post-operative nerve complications except ulnar nerve complications were all radial nerve complications. These four patients were treated using lateral plate longer than 100% of trans-epicondylar distance(TED). It is consistent with result of Wang at el[1].

Of the 11 patients preoperative nerve symptoms, only two patients experienced persistent postoperative radial nerve symptoms. There seems to be no correlation between the preoperative and postoperative nerve symptoms.

### Fixation and osteosynthesis-related complications

Our rates of nonunion (4.1%) were consistent with those reported in the literature. In the literature, excluding two small group studies that reported 0% and 14% [21, 22], fracture nonunion rates were within 2.9%–9.3% [8, 21, 23–25].

One olecranon osteotomy nonunion occurred in 97 cases using the olecranon osteotomy approach. Sotelo et al. reported one olecranon nonunion in five olecranon osteotomies, and Patel et al. reported one olecranon nonunion in 22 olecranon osteotomies [5, 7]. Other studies also reported only one or two olecranon osteotomy nonunions in each study [21, 25].

Mean union time was shortest in parallel plating. But parallel plating didn’t show superiority in nonunion rate. Several biomechanical studies reported parallel plating is more rigid than orthogonal plating. But clinical studies that prove parallel plating is superior than orthogonal plating is still not enough[19, 20, 26, 27].

Two nonunion patients in a total of eight patients with nonunion did not complain of severe pain even though they maintained daily living without a splint and refused osteosynthesis surgery (Fig. 2). Juan et al. [22] reported two nonunion cases in 14 distal humerus transcondylar fracture ORIF, and one case was symptomless nonunion, which is similar to our symptomless nonunion patients.

### Heterotrophic ossification and osteoarthritis

Sotelo et al. identified heterotrophic ossification as a significant factor (*p* < 0.001) of elbow loss of motion [7]. Clavert et al. also found that heterotrophic ossification contributes to the loss of ROM [9]. In our study, heterotrophic ossification significantly decreased flexion ROM. The effect of heterotrophic ossification on extension was not statistically significant. Park et al. identified heterotrophic ossification in the posteromedial aspect of the capsule associated with loss of elbow flexion [28].

Osteoarthritis is a common complication (24.1%). Clavert et al. could not determine whether osteoarthritis was related to extra-articular malunion or intra-articular comminution [9]. The prevalence of degenerative osteoarthritis of the elbow is markedly different, ranging from 2 to 55% [29, 30]. It is difficult to determine whether osteoarthritis is a traumatic or degenerative.

### Complications requiring reoperation

Among the major complications that required urgent obligatory reoperation (22 cases, 12.9%), ulnar nerve complications accounted for half (11 cases).

In our study, instrumentation removal was performed in 59 (34.7%) elbows, and instrumentation removal was performed in 36 (21.2%) asymptomatic patients without special reasons. The cost of instrumentation removal in the study setting is US$ 250, although there is a small variation depending on the hospital tier. Patients are asked to pay 20% of the total cost under our national health insurance service. The low economic burden of instrumentation removal may explain the high removal rates.

The limitations of our study include implant heterogenicity. In 19 years of experience, initially used some plates were not anatomical pre-contoured locking plate. That plates were locking reconstruction plate that bended by surgeon intraoperatively. And this study does note evaluated reduction status using radiologic parameter, although these findings should be meaningful information to predict complication for surgeons.

## Conclusions

After a review of patient data spanning 19 years of experience at multicenter setting, the results of the present study support plate fixation for distal humeral fracture as a reasonable treatment option with acceptable rates of severe complications. Surgeons should be careful when handling the ulnar nerve during ORIF. Prophylactic ulnar nerve anterior transposition is not helpful in preventing postoperative ulnar nerve symptoms (Additional file 1).

## Supplementary Information

**Additional file 1**. Data of all study population of 170 patients.

## Data Availability

All data generated or analyzed during this study are included in this published article and its supplementary information files.

## Abbreviations

ROM: Range of motion
ASH score: Disabilities of arm, shoulder and hand score
ORIF: Open reduction and internal fixation
CRPS: Complex regional pain syndrome
